# Method for B Cell Receptor Enrichment in Malignant B Cells

**DOI:** 10.3390/cancers16132341

**Published:** 2024-06-26

**Authors:** Puja Bhattacharyya, Richard I. Christopherson, Kristen K. Skarratt, Stephen J. Fuller

**Affiliations:** 1 Sydney Medical School Nepean, Faculty of Medicine and Health, The University of Sydney, Penrith, NSW 2750, Australia; pbha4047@uni.sydney.edu.au (P.B.); kristy.skarratt@sydney.edu.au (K.K.S.); 2 Blacktown Hospital, Blacktown Rd., Blacktown, NSW 2148, Australia; 3 School of Life and Environmental Sciences, University of Sydney, Sydney, NSW 2006, Australia; richard.christopherson@sydney.edu.au; 4 Nepean Hospital, Derby Str., Kingswood, NSW 2747, Australia

**Keywords:** B cell, B cell receptor, enrichment, mass spectrometry

## Abstract

**Simple Summary:**

The B cell receptor (BCR) is a membrane-bound protein complex that is required for the normal development of B cells. BCR signalling is also involved in the pathogenesis of B cell cancers. While there is substantial literature on genomic analyses of the BCR, there are limited proteomic studies of receptor structure and its interactions with neighbouring proteins. This is partly due to the location of the BCR in the surface-membrane lipid environment that has limited the ability to enrich the complex for proteomic analysis. Here, we report an enrichment technique that can be used for mass spectrometry analyses of the BCR from live B cells.

**Abstract:**

B cells are central to the adaptive immune response and provide long-lasting immunity after infection. B cell activation is mediated by the surface membrane-bound B cell receptor (BCR) following recognition of a specific antigen. The BCR has been challenging to analyse using mass spectrometry (MS) due to the difficulty of isolating and enriching this membrane-bound protein complex. There are approximately 120,000 BCRs on the B cell surface; however, depending on the B cell activation state, there may be hundreds-of-millions to billions of proteins in a B cell. Consequently, advanced proteomic techniques such as MS workflows that use purified proteins to yield structural and protein-interaction information have not been published for the BCR complex. This paper describes a method for enriching the BCR complex that is MS-compatible. The method involves a Protein G pull down on agarose beads using an intermediary antibody to each of the BCR complex subcomponents (CD79a, CD79b, and membrane immunoglobulin). The enrichment process is shown to pull down the entire BCR complex and has the advantage of being readily compatible with further proteomic study including MS analysis. Using intermediary antibodies has the potential to enrich all isotypes of the BCR, unlike previous methods described in the literature that use protein G-coated beads to directly pull down the membrane IgG (mIgG) but cannot be used for other mIg isotypes.

## 1. Introduction

**Figure 1:**
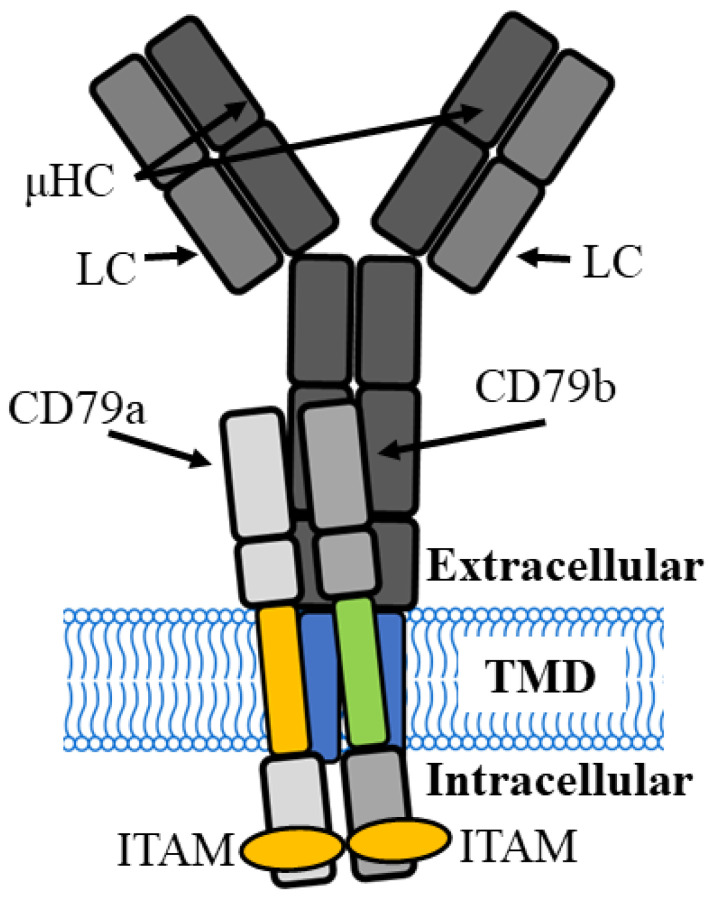
B cell receptor complex of the immature B cell has two µ heavy chains (µHC) combined with two light chains (LC) to form a membrane-bound, antigen-binding immunoglobulin M (IgM) that interacts with a CD79a and CD79b signalling module. TMD, transmembrane domain, ITAM, immunoreceptor tyrosine-based activation motifs.

B cells are critical to the adaptive immune response and provide long-lasting immunity after infection [1]. At different developmental stages, B cell survival, activation, proliferation and differentiation are controlled by the B cell receptor (BCR) complex, which is a membrane-bound version of one of its five secreted immunoglobulin isotypes (mIgM, mIgD, mIgA, mIgE, and mIgG) consisting of two identical, disulfide-linked heavy and light chains that form a tetramer bound to a heterodimer made up of Igα (CD79a) and Igβ (CD79b) [2,3]. The extracellular component of the mIg binds antigen, and signalling into the cell occurs through the CD79a/CD79b heterodimer. The mIg forms a “Y” shape with two fragment antigen binding (Fab) domains facing out from the surface membrane into the extracellular space and a fragment crystallizable region (Fc) tail that is inserted into the surface membrane. Each light chain is encoded by a gene locus that contains a constant (C) region and sets of variable (V) and joining (J) segments, whereas the heavy chain, in addition, contains a set of diversity (D) segments. During B cell development, these segments are assembled by site-specific recombination that is coordinated by a V(D)J recombinase. Random recombination of the variable regions generates an enormous range of antigen-binding specificities that are unique to each individual BCR [4]. Antigen binding to the Fab region results in signal transduction to the Fc region and, within the surface membrane, signal transmission to the CD79a/79b complex and signal transduction via the cytoplasmic tails to the interior of the B cell and to the nucleus, via intracellular signalling events (Figure 1). This culminates in the transcription of genes that regulate B cell activation, proliferation, survival, and cytoskeletal re-organisation that is required to generate an immune response [5]. Following antigen stimulation in peripheral lymphoid organs, with the help of activation-induced deaminase and helper T cell-dependent processes, immunoglobulin (Ig) genes undergo somatic hypermutation and class-switch recombination that produces high-affinity IgG, IgA and IgE, which make up the secondary Ig repertoire to accompany IgM and IgD. Each heavy chain is combined with one of two light chains, kappa (κ) or lambda (λ) [3].

BCR signalling is regulated by interaction with other proteins, including CD19 and FcγRIIB [6,7], and the earliest events that occur following initiation of signalling after antigen binding to the BCR are highly sensitive to the affinity of the BCR for antigen [8]. In both the immature and mature B cell, initiation of signalling and signal transmission to the CD79a/79b complex are pivotal points in the initiation and modulation of downstream BCR signalling. The location of this interaction between the mIg and CD79a/CD79b is in the lipid environment of the surface membrane that has hindered proteomic analysis of both the structure and movement of the BCR transmembrane domain (TMD) after antigen binding.

Binding of antigen to the BCR leads to the formation of surface-membrane BCR microclusters that activate intracellular tyrosine kinases. BCR microclusters and associated proteins generate signals that depend on the number and/or affinity of the antigen, suggesting that microclusters are an elementary signalling unit. However, the structure of microclusters and proteins that drive their formation are not known.

Aberrant signalling through the BCR and its co-receptors promotes the pathogenesis of several B cell malignancies and autoimmune diseases [2]. Malignancies harness and adapt BCR signalling pathways and recruitment of accessory cells such as T helper cells in the tissue microenvironment that promote clonal growth and survival [9]. Compared to normal, non-malignant B cells, the BCR in lymphomas and leukaemias may have markedly different cell-surface expressions and responses to a wide range of foreign and self-antigens [10].

BCR signalling is critical in determining B cell outcomes at several checkpoints [11]. These checkpoints prevent the survival of B cells that fail to express a functional heavy- and light-chain pair, limit the numbers that carry two different heavy chains, and remove autoreactive BCR combinations. Although additionally regulated by co-stimulatory and antagonist receptors, outcomes at these checkpoints rely on the BCR to physically signal in different ways. Critically, this signalling intersects at the interface between the CD79a/CD79b heterodimer and the heavy-chain homodimer within the B cell surface membrane. Understanding how the signalling module interacts with the antigen-binding module of the BCR and ultimately how ligand binding is transformed into pro-survival, apoptotic and receptor editing signals is essential to understanding the role of the BCR in health and disease. Like other membrane-spanning proteins, the BCR contains hydrophobic TMDs and hydrophilic extra- and intra-cellular domains that create significant challenges for solubilization, purification and studying functionally active forms of the receptor. The implications of developing a method for BCR enrichment are far-reaching and would be applicable to many advanced proteomic techniques, of which cross-linking mass spectrometry (XL-MS) is an example. XL-MS has been developed to stabilise protein complexes before they are degraded by cell lysis and protein purification methods. The results are more biologically relevant as the target protein is purified based on its natural expression and conformation. However, the complexity of samples generated by whole-cell cross-linking has limited XL-MS to identifying abundant proteins that contain many cross-links.

In this paper, we report a targeted purification method using anti-BCR antibodies and beads to purify the receptor from cell lysates. The aim of this method would be to produce an enriched BCR product in sufficient quantities for advanced proteomic analysis, and one such application includes XL-MS. Consequently, the method was adapted so that the enrichment steps and final solubilization could be undertaken in an MS-compatible detergent. Current enrichment strategies often modify the BCR from its endogenous state by using transfected cell lines, recombinant proteins and tags before transient protein interactions can be captured. There is an unmet need to improve techniques to enrich and isolate the BCR for traditional and novel MS techniques. Our study will make a valuable contribution to the methodology for such techniques in native B cells by enriching the BCR in sufficient quantities to harvest for this technique and will be broadly applicable to other membrane-bound proteins. This paper describes a method to directly pull down the BCR of mIg isotypes in sufficient quantity and purity for further proteomic analysis including MS studies.

## 2. Materials and Methods

### 2.1. General Consumables

Triton X-100, phosphate buffered saline (PBS), sodium deoxycholate (DOC), DL-dithiothreitol (DTT), bovine serum albumin (BSA), HEPES (4-(2-hydroxyethyl)-1-piperazineethanesulfonic acid), potassium chloride (KCl), glycine, Tris buffered saline (TBS), Tween 20, RPMI-1640, 100× penicillin/streptomycin and methanol were all sourced from Sigma-Aldrich Pty. Ltd.(Castle Hill, Australia) Fetal bovine serum (FBS) was from Thermo Fisher Scientific Australia Pty. Ltd., Scoresby, Australia. All solutions were prepared using Milli Q water (MQ, resistivity > 18.2 MΩ·cm).

The following antibodies against specific components of the BCR were used in pull-down assays: murine anti-human CD79a IgG1, (Santa Cruz, # 390372; 200 μg/mL), murine anti-human IgM IgG2b, (Santa Cruz, # 69920; 100 μg/mL), and rabbit anti-human CD79b IgG, (Abcam # ab134147; 102 μg/mL). Santa Cruz antibodies were sourced from Bio Strategy Pty. Ltd., Melbourne, Australia.

The secondary antibodies were the following: donkey anti-mouse IR680 conjugate (LI-COR catalogue number: LCR-925-68072) or donkey anti-rabbit CW800 (LI-COR, # LCR-925-32213). Rabbit recombinant monoclonal IGHG1 antibody (Abcam # ab181236) was used to probe OSU chronic lymphocytic leukaemia (CLL) membrane fractions.

### 2.2. Methods

#### 2.2.1. Cell Preparation

The Raji human B lymphoblastoid cell line was obtained from the American Type Culture Collection (ATCC, number CCl-86) and the OSU-CLL cell line was obtained under a material transfer agreement from Dr Oliver Giles Best, University of Sydney. Both cell lines were maintained in RPMI-1640 medium supplemented with 10% FBS, 2 mM L-glutamine, 100 U/mL penicillin, and 100 μg/mL streptomycin at 37 °C with 5% CO_2_ in a humidified incubator. The Raji cell line was used to establish the method as described in the results. The OSU cell line was used to confirm that the pull-down method was reproducible in a second cell line. Cells were grown to confluence (approximately 1 × 10^6^ cells/mL) and approximately 25 × 10^6^ cells were used for each enrichment experiment.

#### 2.2.2. Preparation of Whole-Cell Lysates

A 4× stock solution of protease inhibitor (PI) cocktail was freshly prepared by dissolving one complete ultra mini, EDTA-free protease inhibitor tablet (Sigma-Aldrich Pty. Ltd., Roche Catalogue # 5892791001) in MQ (2.5 mL). The cells were harvested by centrifuging at 300× *g* for 5 min at 4 °C and washed three times in cold PBS (5 mL each wash, centrifuging as above). The residual cell pellet was re-suspended in 4–5 volumes of Triton X-100 Lysis buffer (1% Triton X-100, 2 × PI in PBS). The suspension was vortexed and incubated on ice for 1 h. The final suspension was centrifuged at 16,000× *g* for 10 min at 4 °C and the whole-cell lysate (WCL) was collected for analysis.

#### 2.2.3. Separation of Membrane Fraction from Cytosol

**Figure 2:**
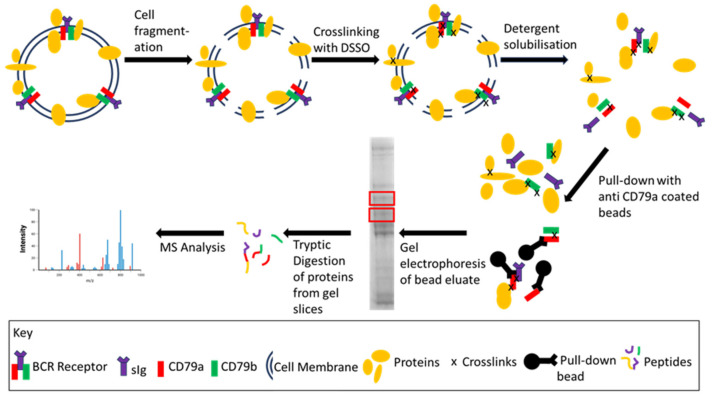
General workflow of BCR enrichment coupled with an example of downstream proteomic analysis. Following cell lysis/fragmentation, the BCR and associated proteins were cross-linked with DSSO to stabilize protein–protein interactions. The membrane was solubilized using either Triton-X 100 or DOC and the BCR was enriched using anti-CD79a coated beads. The BCR and associated proteins are eluted from the beads, separated by gel electrophoresis and gel slices (red boxes) are analysed by MS.

Cells were harvested and washed 3 times in ice-cold PBS (5 mL) and centrifuged at 300× *g* for 5 min at 4 °C. The residual pellet was suspended for 5 min at 4 °C in 500 µL of hypotonic lysis buffer (10 mM HEPES, 15 mM KCl, and 2 × PI in MQ) to swell the cells before snap freezing in liquid nitrogen. Frozen cells were thawed rapidly, and the freeze/thaw procedure was repeated to induce cell lysis (Figure 2). The thawed suspension was centrifuged (16,000× *g* for 10 min at 4 °C) to separate the cytosolic fraction (supernatant) from the membrane fraction (pellet).

The pellet was washed three times with hypotonic lysis buffer (1 mL) by centrifugation (16,000× *g* for 5 min at 4 °C). To solubilize the membrane proteins, the pellet was incubated for 60 min at 4 °C either in Triton X-100 lysis buffer (described above) or with varying concentrations of DOC buffer (0.25%, 0.5%, or 1% DOC) with 2 × PI in 10 mM Tris pH 7.5. The suspension was centrifuged as above, and the supernatant collected for analysis.

The protein concentrations of samples in Triton X-100 lysis buffer were determined using Bio-Rad Protein Assay Dye Reagent (# 500006) to perform a Bradford Assay [12] in a 96-well plate. For compatibility with the dye reagent, samples were diluted 1 in 10 in MQ prior to the assay to reduce the Triton X-100 concentration from 1% to 0.1%. Samples and standards were prepared in triplicate and absorbances at 595 nm were read on a Fluostar Optima plate reader (BMG Labtech Pty. Ltd., Melbourne, Australia). For samples in DOC buffer, protein concentrations were determined by measuring absorbance at 280 nm using a NanoDrop 2000 Spectrophotometer (ThermoFisher Pty. Ltd., Riverstone, Australia).

#### 2.2.4. Enrichment by Pull Down Using Protein G Agarose Beads

Antibodies against specific components of the BCR were used in pull-down assays (Figure 2). The solubilised membrane fraction was incubated with each pull-down antibody for 2 h with gentle mixing on a rotator at 4 °C. The protein-load-to-antibody concentration depended on the specific experiment, as described in the results. Aliquots of Protein G pull-down beads (EZview Red Protein G Affinity Gel Sigma-Aldrich Pty. Ltd., # E3403) were prepared by washing three times in 1 mL of the corresponding solubilisation buffer (either Triton X-100 lysis buffer or DOC lysis buffer) and spun at 8500× *g* for 1 min at 4 °C. The mixture of antibody and solubilized cell membrane was then added to the prepared beads and incubated overnight at 4 °C with gentle mixing on the rotator. In later experiments, the WCL was incubated with the antibodies, followed by addition of protein G beads to determine if the sequence of pull down improved enrichment of the BCR.

Following incubation, the beads were washed 3x with solubilisation buffer, as above. The attached protein was eluted from the beads by suspending in 1× NuPage™ LDS sample buffer (20 µL) with 100 mM DTT, and heated at 70 °C for 10 min. The suspension was centrifuged at 16,000× *g* for 10 min at RT and the supernatant containing the released proteins was collected for gel electrophoresis.

#### 2.2.5. Gel Electrophoresis and Protein Transfer

The WCL, membrane and cytosolic fractions were prepared for electrophoresis by heating protein (30 µg) at 70 °C for 10 min in 1× NuPage™ LDS sample buffer with 100 mM DTT. After vortexing, samples and molecular-weight (MW) markers (Prestained Protein Ladder–Broad MW 10–245 kDa; Abcam ab116028 or Novex^®^ Sharp Pre-stained Protein Standard, Thermo Fisher #LC5800) were loaded onto a precast 7% NUPAGE Tris-Acetate Gel (Thermo Fisher., # EA0355BOX) and run for 70 min in Tris acetate sodium dodecyl sulfate (SDS) running buffer (50 mM Tricine, 50 mM Tris base, 0.1% SDS pH 8.24) in a Mini Gel Tank (Thermo Fisher, # A25977) at 150 V.

After electrophoresis, the gels were incubated for 5 min in cold Towbin’s transfer buffer (25 mM Tris, 192 mM glycine, 10% methanol, pH 8.3). Immobilon^®^-FL PVDF membranes (Merck Millipore # IPL00010) were prepared for transfer by soaking in methanol for 30 s before incubating in the transfer medium for 5 min. Transfer was performed in a wet transfer tank (Bio-Rad Mini Trans-Blot Cell # 1703930) at 350 mA for 50 min at RT.

After transfer, protein loading was assessed using a TotalStain Q (PVDF) kit (Azure Biosytems Inc., Dublin, CA, USA # AC2225) following the manufacturer’s instructions. Briefly, the membrane was rinsed with MQ and stained for 5 min then washed 3 times in the rinse buffer supplied in the kit before images were captured using the total protein Q pre-set program in the Azure 500Q imager. The blot was washed for 5 min in MQ before Western blotting.

#### 2.2.6. Western Blotting

##### Near Infra-Red Blots (NIR)

Membranes were blocked using Intercept™ (TBS) Blocking Buffer (IBS) (Li-Cor # 927-60001) at RT for 1 h with gentle agitation, then washed three times in 10 mL of 0.1% Tween 20 in TBS (TBS-T) for 5 min. The same antibodies targeting the components of the BCR in pull-down assays were used as primary antibodies for Western blotting (Figure 2). Primary antibodies were diluted to between 1 in 200 and 1 in 10,000 in IBS (10 mL) and incubated with the membrane by gentle agitation overnight at 4 °C. The membrane was washed four times in TBS-T (10 mL) for 5 min with gentle agitation. The secondary antibodies [donkey anti-mouse IR680 conjugate) or donkey anti-rabbit CW800 conjugate were diluted to 1:10,000 by adding 1 µL to 10 mL of intercept blocking buffer/TBS and 100 µL of 0.1% SDS. Membranes were incubated for 1 h at RT in the dark before washing three times with TBS-T as above, in the dark. Finally, the blot was rinsed in water before drying. NIR images of the dried blots for each conjugate were collected simultaneously, using an Azure 500Q imaging system.

##### Chemiluminescent Blots

Chemiluminescence was used for visualizing the IgM component of the BCR by Western blot. The chemiluminescent blots were incubated with 5% BSA in TBS-T for 1 h with gentle agitation at RT and washed three times in TBS-T (10 mL) for 5 min with gentle agitation. Murine anti-human IgM was diluted to between 1 in 200 and 1 in 1000 in TBS-T and incubated with the membrane with gentle agitation overnight at 4 °C. The membranes were then washed four times in TBS-T (10 mL) for 5 min with gentle agitation. Goat anti-mouse HRP (Abcam Australia Pty Ltd. Goat Anti-Mouse IgG (H+L)-HRP conjugate # ab6789) was diluted 1 in 10,000 in TBS-T and incubated with the membrane for 1 h at RT with gentle agitation before washing, as above. Blots were developed as described by Mruk and Cheng [13]. Briefly, blots were incubated in chemiluminescent development buffer (10 mL, 0.1 M Tris pH 8.6 at 22 °C with 1.25 mM luminol, 0.2 mM p-coumaric acid and 3 µL of 30% *w*/*v* hydrogen peroxide) added immediately before incubating the blot for 1 min. An image was captured using the Azure 500Q imager with the Chemi Blot module.

Image J software version 1.54 h was used to quantify total protein load in each lane by first inverting the colours and then calculating the area under the curve which was representative for each lane. The corresponding bands for the CD79a, CD79b and IgM lanes were quantified in a similar manner. The result for each blot was represented as a ratio normalized to the protein concentration of each lane, to allow meaningful comparison across lanes.

#### 2.2.7. Crosslinking and In-Gel Digest Mass Spectrometry

As a demonstration of the potential applications of the enrichment process, with a specific focus on in-gel digest MS techniques, preliminary studies were undertaken using cross-linking mass spectrometry with commercially available disuccinimidyl sulfoxide (DSSO) (Cayman Chemical, #9002863) combined with the optimized enrichment technique described (Figure 2).

In brief, the method was adapted from Bartolec et al. and Klykov et al. [14,15] and uses the hypotonic lysis technique with suspension of the cells in HLB and freeze/thawing described above. A stock solution of 50 mM DSSO in dimethyl sulfoxide was prepared and was immediately added to the lysed cell suspension to a final concentration of 5 mM and incubated at RT for 30 min. The crosslinking reaction was quenched by adding TRIS to a final concentration of 10 mM and incubating for 15 min. The cytosolic fraction was separated from the membrane fraction as described previously, and the membrane-bound proteins were solubilized with the MS-compatible detergent 0.5% DOC lysis buffer. Agarose bead pull-down enrichment was undertaken as described above, using anti-CD79a antibody, and the bead eluate was subjected to SDS PAGE (as described above). Protein bands were visualized using Coomassie Blue staining [16].

High-MW bands visualized in the Coomassie stain were excised. The gel plugs were destained using destain solution (40% *v*/*v* acetonitrile in 12 mM ammonium bicarbonate). Destain solution (40 µL) was added to the gel plug and shaken at RT for 10 min and the destain solution was decanted. The process was repeated 2–4 times until the Coomassie dye was removed. The gel plugs were then reduced with 5mM DTT in 50 mM ammonium bicarbonate for 40 min followed by alkylation with 10 mM iodoacetamide (IAA) in 50 mM ammonium bicarbonate with 0.5% DOC for 30 min in the dark before being rinsed in 100% acetonitrile. The gel pieces were dried in a Speed-Vac Concentrator (Thermo Fisher Scientific) for 5 min. The gel pieces were digested in a volume of trypsin sufficient to fully immerse the gel pieces (3–15 µL of 12 ng/µL trypsin in 50 mM ammonium bicarbonate; Promega Corp, # V5117) and incubated at 4 °C for 1 h before excess trypsin was removed and the gel was immersed in 50 mM ammonium bicarbonate and incubated at 37 °C overnight. Formic acid was added to a final concentration of 1% to inactivate the enzymatic reaction. The supernatant was pipetted off and saved in the final analysis tube. To the gel pieces in the original tube, 30 µL of 50% acetonitrile with 5% of formic acid was added to each sample and incubated for 45 min, followed by sonication for 5 min. The supernatant from this solution was again transferred and added to the final analysis tube. The procedure was repeated once more using 30 µL of 90% acetonitrile with 5% formic acid and incubated for 5 min with sonication of the original gel pieces. The supernatant was removed and added to the final analysis tube. The supernatant in the final analysis tube was dried to completion, using a speed vacuum. The dried peptides were resuspended in approximately 5 µL of loading buffer (0.1% formic acid/3% acetonitrile), ready for MS input.

High-MW bands visualized in the Coomassie stain were excised. The gel plugs were destained using destain solution (40% *v*/*v* acetonitrile in 12 mM ammonium bicarbonate). Destain solution (40 µL) was added to the gel plug and shaken at RT for 10 min and the destain solution was decanted. The process was repeated 2–4 times until the Coomassie dye was removed. The gel plugs were then reduced with 5mM DTT in 50 mM ammonium bicarbonate for 40 min followed by alkylation with 10 mM iodoacetamide (IAA) in 50 mM ammonium bicarbonate with 0.5% DOC for 30 min in the dark before being rinsed in 100% acetonitrile. The gel pieces were dried in a Speed-Vac Concentrator (Thermo Fisher Scientific) for 5 min. The gel pieces were digested in a volume of trypsin sufficient to fully immerse the gel pieces (3–15 µL of 12 ng/µL trypsin in 50 mM ammonium bicarbonate; Promega Corp, # V5117) and incubated at 4 °C for 1 h before excess trypsin was removed and the gel was immersed in 50 mM ammonium bicarbonate and incubated at 37 °C overnight. Formic acid was added to a final concentration of 1% to inactivate the enzymatic reaction. The supernatant was pipetted off and saved in the final analysis tube. To the gel pieces in the original tube, 30 µL of 50% acetonitrile with 5% of formic acid was added to each sample and incubated for 45 min, followed by sonication for 5 min. The supernatant from this solution was again transferred and added to the final analysis tube. The procedure was repeated once more using 30 µL of 90% acetonitrile with 5% formic acid and incubated for 5 min with sonication of the original gel pieces. The supernatant was removed and added to the final analysis tube. The supernatant in the final analysis tube was dried to completion, using a speed vacuum. The dried peptides were resuspended in approximately 5 µL of loading buffer (0.1% formic acid/3% acetonitrile), ready for MS input.

For XL-MS studies, the mass spectrometry parameters for crosslinking analysis were adapted from Klykov et al. [15]. The instrument used was an Orbitrap (OT) Eclipse Tribrid mass spectrometer (ThermoFisher Scientific). Firstly, in the MS1 stage, a survey scan was recorded for simple identification of the peptides. For the selected precursors, collision-induced dissociation (CID) was used and the signature peaks for the cross-linkers were recorded in the middle resolution (MS2). Fragments demonstrating patterns associated with DSSO were selected and then further fragmented to a low resolution in the MS3 scan in the ion trap (IT) [15]. For membrane-bound proteins, cross-linking required further optimisation and therefore only MS1 data are shown (see Table S1 for detailed instrument settings). Bovine serum albumin (BSA) was used as the control for the system suitability test (SST), which demonstrated the liquid chromatography–mass spectrometry (LC–MS) was running to the required specification. For further XL-MS characterization, we planned to use a CID—electron transfer with supplemental activation by higher-energy collision dissociation (EThcD)–MS2—higher-energy collisional dissociation (HCD)–MS3 (CID-EThcD-MS2-HCD-MS3) protocol. Data analysis was undertaken using Proteome Discoverer (PD-Thermo Fisher Scientific) to identify peptides in each gel slice (workflow described in Supplementary Table S2.

## 3. Results

### 3.1. Confirmation That the Raji Cell Line Has All Components of the BCR

A blot was prepared using Raji WCL and cut into strips that were probed with anti-CD79a (1/500 dilution), anti-CD79b (1/1000 dilution) and anti-IgM (1/500 dilution) and visualised as described in the methods. A band corresponding to the expected MW of each antibody target was detected, confirming that the Raji cell line expresses all three components of the BCR and that the Raji cell line expresses IgM (Figure S1).

Blots were prepared using Raji WCLs to optimise the concentrations of primary antibodies for the BCR components. Before blocking, total protein loading was determined as described in the methods. After blocking, blots were cut into individual lanes and each lane was incubated with a single concentration of primary antibody and washed separately. All the strips were then incubated with the same secondary antibody solution, and visualized together. Detected protein was normalized to the total protein load (Figure S2). The optimal primary-antibody concentration was determined as the concentration that produced the most distinct band whilst avoiding non-specific staining that was seen as additional bands (Figure S2). The optimal concentrations for subsequent experiments were the following: 1/500 for anti-CD79a, 1/500 for anti-IgM and 1/1000 for anti-CD79b.

**Figure 3:**
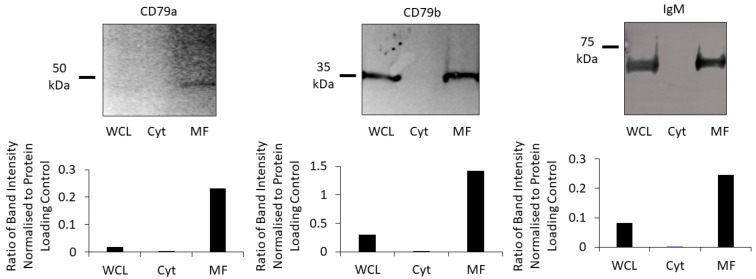
Proportion of each subunit of the BCR in cell fractions. CD79a, CD79b and IgM were identified using Western blot (upper row) in the WCL (lane 1), the cytosolic fraction (Cyt; lane 2), and the membrane fraction (MF; lane 3). Bar chars (lower row) show the ratios of each subunit normalised to total protein loaded in the WCL, Cyt and MF for each Western blot.

To confirm that components of the BCR were localized to the membrane, Western blotting was performed using WCL, the cytosolic fraction and the membrane fraction (solubilized in Triton X-100 lysis buffer) from the same batch of Raji cells. All three components of the BCR were detected in the WCL and the membrane fraction, with little-to-none detected in the cytosolic fraction (Figure 3). Therefore, the membrane fraction was used for subsequent experiments.

### 3.2. Pull-Down Enrichment of the BCR with Protein G Beads

**Figure 4:**
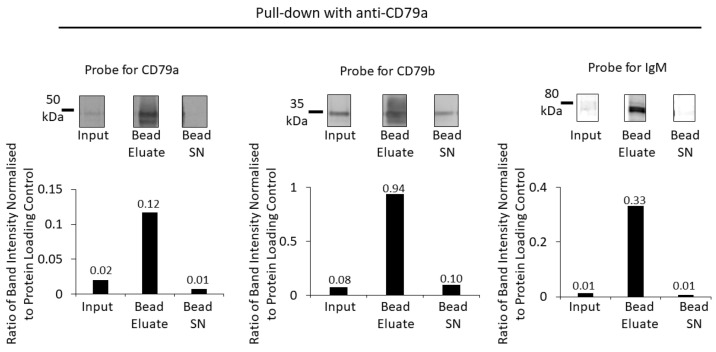
Enrichment of BCR components using pull down with anti-CD79a and protein G beads. Anti-CD79a was used to pull down proteins from a Raji membrane fraction WCL. A Western blot of the membrane fraction (input), bead eluate and the bead supernatant (Bead SN) was probed with antibodies against CD79a, CD79b and IgM (top row). Bar charts (bottom row) show the ratios of Western-blot band intensities normalized to protein-loading control for CD79a, CD79b and IgM. Numerical values of the ratios are indicated on the bar charts.

To determine if all components of the BCR could be pulled down using anti-CD79a antibody, 5 μL (1 μg) of antibody was pre-incubated with 500 µg of the membrane fraction before overnight incubation with protein G beads, as described in the Methods section. Western blots of the membrane fraction (input), bead eluate and bead supernatant were prepared. After determining the total protein load, the membrane was probed with anti-CD79a and anti-CD79b and visualized using NIR secondary antibodies. The same membrane was then probed with anti-IgM and visualized using the chemiluminescent method described (Figure 4).

The bead eluate from the pull down with anti-CD79a showed a high-intensity band corresponding to the MW of CD79a that was six-fold the intensity of the input band intensity when normalized for protein loading (Figure 4). A minimal amount of CD79a was detected in the bead supernatant, indicating that the majority of CD79a had been captured by the pull-down process (Figure 4). When the CD79a pull-down bead eluate was analysed for co-precipitation of CD79b, an approximately 12-fold enrichment of CD79b was found; however, a substantial proportion remained in the bead supernatant (Figure 4). Pull down with anti-CD79a achieved an enrichment of IgM (~30 fold), with minimal IgM remaining in the supernatant.

**Figure 5:**
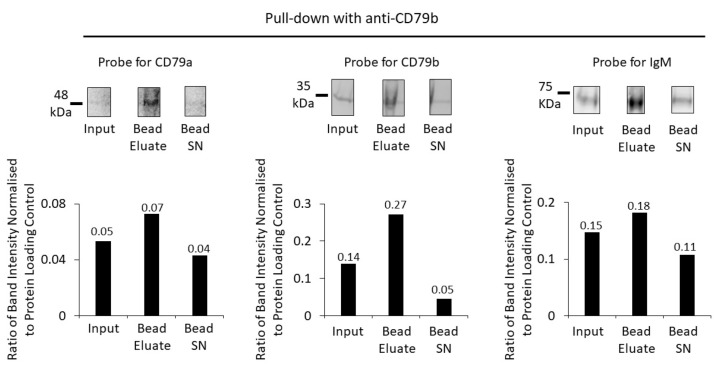
Enrichment of BCR components using anti-CD79b and protein G beads. A Western blot of the membrane fraction (input), bead eluate, and the bead supernatant (Bead SN) was probed with antibodies against CD79a, CD79b and IgM (top row). Bar charts (bottom row) show the ratios of Western-blot band intensities normalized to protein-loading control for CD79a, CD79b and IgM. Numerical values of the ratios are indicated on the bar charts.

**Figure 6:**
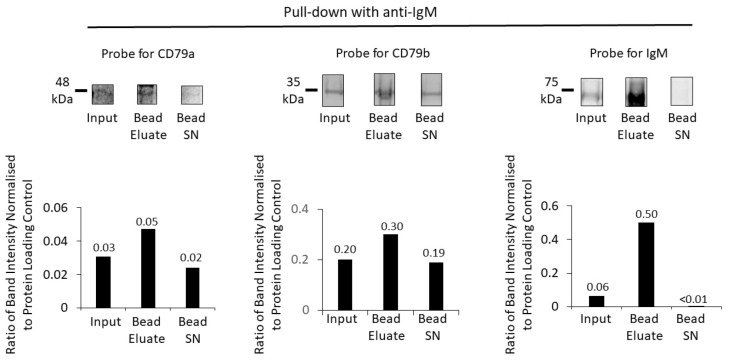
Enrichment of BCR components using anti-IgM and protein G beads. A Western blot of the membrane fraction (input), bead eluate, and the bead supernatant (Bead SN) was probed with antibodies against CD79a, CD79b and IgM (top row). Bar charts (bottom row) show the ratios of Western-blot band intensities normalized to protein-loading control for CD79a, CD79b and IgM. Numerical values of the ratios are indicated on the bar charts. Protein fractions were loaded on the same gel and sequentially analysed on the same membrane, first with NIR analysis of CD79a and CD79b and followed by chemiluminescent detection of IgM.

BCR enrichment was replicated using anti-CD79b (Figure 5) and anti-IgM to pull down the receptor from the membrane fraction (Figure 6). Pull down with anti-CD79b showed some enrichment of the target protein but minimal enrichment of either CD79a or IgM (Figure 5).

Similarly, for pull down with anti-IgM, this effectively pulled down IgM, together with CD79a and CD79b (Figure 6). 

There was pull down and enrichment of all BCR components, most markedly for the target protein IgM (with an approximately 8-fold increase). Compared to anti-CD79a pull down, however, there was less effective co-immunoprecipitation (IP) of the other two components of the BCR, CD79a and CD79b.

These experiments demonstrated that pull down with anti-CD79a was the most effective method for enriching the entire BCR complex compared to anti-CD79b or anti-IgM, and anti-CD79a was used as the pull-down antibody for subsequent experiments.

The pull-down process with anti-CD79a antibody was replicated using a second B cell line (OSU B-CLL cell line), showing that the method can be applied in different B cell lines (Figure S3).

### 3.3. Optimising Pull-Down Antibody Concentration

The optimal concentration of anti-CD79a was then determined (Figure S4). Raji cell membrane fractions (500 µg of protein in 480 µL of Triton X-100 lysis buffer) were incubated with anti-CD79a antibody: 2.5 µL (0.5 µg), 5 µL (1 µg), or 10 µL (2 µg). In a Western blot, the intensity of the bands for CD79a and CD79b in the bead eluate (expressed as a normalized ratio of the protein-loading control), was highest for 5 µL of anti CD79a to 500 µg of input membrane fraction. The protein-loading control was adjusted to reflect differences in the protein load from the pull-down antibody.

### 3.4. Strategies to Improve BCR Enrichment

To improve BCR enrichment, variations in the bead washing procedure (Figure S5) and the order of anti-CD79a antibody application in the pull-down assay (Figure S6) were studied.

The procedure for washing the beads after overnight incubation was made more stringent by including a 5 min incubation with gentle mixing on the rotator at 4 °C as part of each wash. As previously, 500 µg aliquots of membrane fraction were incubated with 5 µL of anti-CD79a antibody before adding protein G beads for overnight incubation. Beads were washed with standard and stringent protocols. Western blots were prepared from the bead eluates, subjected to each wash procedure, and probed for CD79a and CD79b (Figure S5).

The more stringent wash procedure lowered the yield but enriched both the target protein and the co-immunoprecipitated CD79b approximately 2-fold.

The order of addition of the antibody in the pull-down assay was then tested. For the standard procedure, 5 µL of anti-CD79a was incubated with 500 µg of WCL for 2 h at 4 °C before adding the protein G beads. In the alternative procedure, 5 µL of antibody was incubated with the protein G beads for 2 h at 4 °C before adding 500 µg of membrane fraction. Both samples were incubated overnight, and bead eluates were collected using the standard washing procedure, and Western blots were prepared and probed using anti-CD79a and anti-CD79b (Figure S6). Greater enrichment was achieved by preincubation of protein G beads with the pull-down antibody before adding the membrane-fraction protein compared to incubating the membrane fraction with the antibody before adding the beads (Figure S6).

### 3.5. Demonstrating the Effectiveness of DOC, an MS-Compatible Detergent

To remove the BCR from the lipid surface membrane for MS analysis, DOC, an MS-compatible detergent was used [17,18,19,20], instead of Triton X-100, which is MS-incompatible [20]. CD79a and CD79b were identified using Western blotting after anti-CD79a antibody pull down from the membrane fraction. The optimal concentration was determined using serial dilutions. Use of 1% DOC buffer resulted in a viscous suspension that could not be loaded onto the gel, and consequently, 0.25%- and 0.5%-DOC buffers were used (Figure S7). The Western-blot band intensities normalised for protein loading were highest when using 0.5% DOC buffer; therefore, this buffer concentration was used for subsequent experiments.

**Figure 7:**
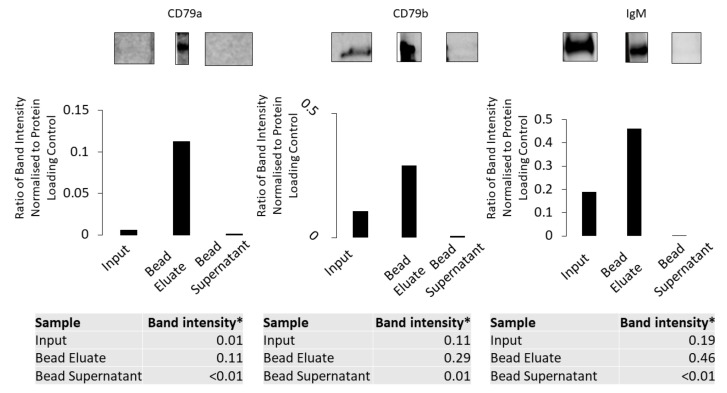
Anti-CD79a antibody pull down using the membrane fraction solubilized in 0.5% DOC buffer. CD79a, CD79b and IgM were detected using Western blotting in the membrane fraction, bead eluate and bead supernatant. All three components are enriched in the bead eluate. * Ratio of band intensity normalised to protein-loading control.

Using 0.5% DOC buffer it was shown that anti-CD79a pull down from the membrane fraction enriches all components of the BCR (Figure 7).

### 3.6. Preliminary Evidence That BCR-Enrichment Process Is Applicable to Advanced Proteomics Technique: XL-MS

Having established an effective method for BCR enrichment demonstrable by increased intensity of the respective bands corresponding to all the BCR subcomponents in the bead eluate on Western blot, we wanted to show preliminary evidence that this method was applicable to advanced proteomics techniques. We selected XL-MS as a powerful tool to study protein interactions to trial our BCR-enrichment technique developed in this paper.

**Figure 8:**
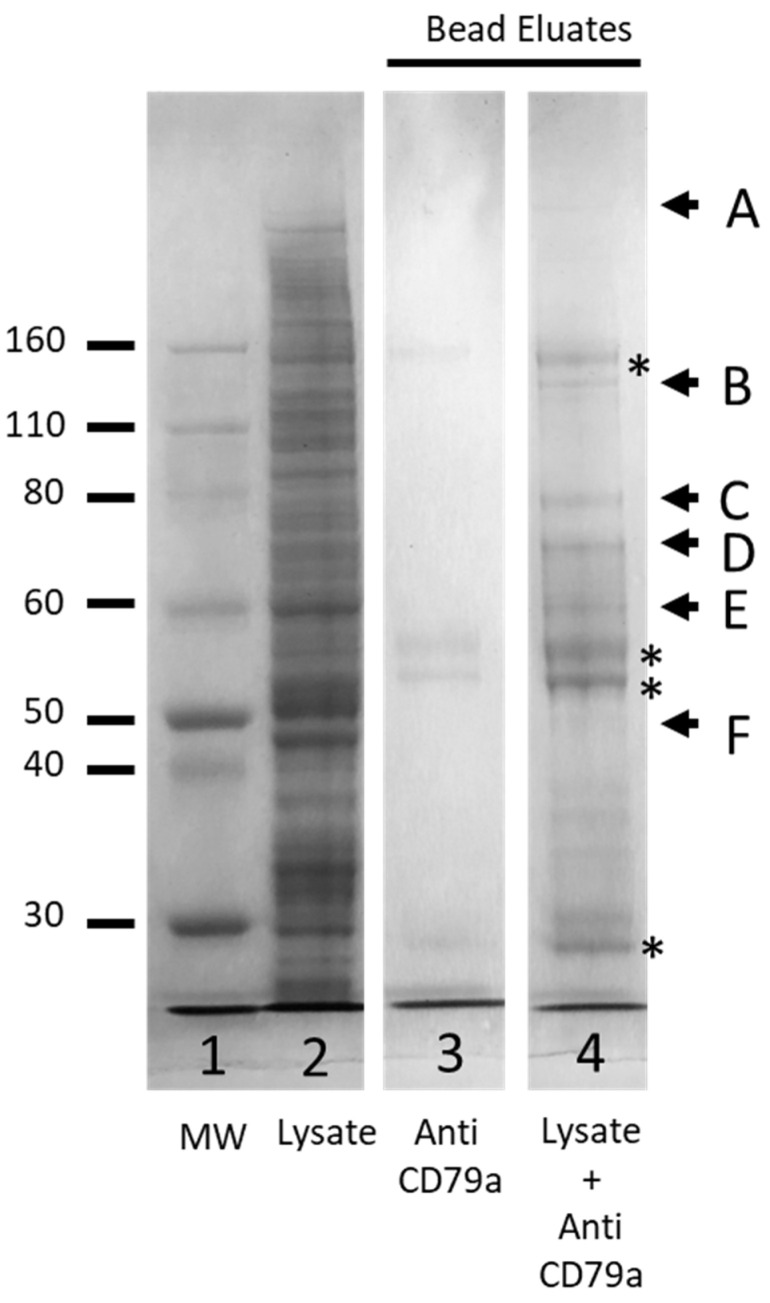
Coomassie-stained SDS-PAGE analyses of crosslinked membrane fraction of Raji cell line enriched by anti-CD79a pull down. Lane 1, protein ladder (MW, Thermo Fisher); Lane 2, crosslinked membrane fraction, (MF); Lane 3, eluate from beads incubated with anti CD79a alone; Lane 4, eluate from beads incubated with MF and anti-CD79a. Bands marked A–F were excised for MS analysis. * Indicates bands associated with anti-CD79a pull-down antibody.

DSSO is a crosslinker that can be used in MS techniques to interrogate protein structure and interactions between proteins. To investigate the feasibility of using this technology to study the structure of the BCR we crosslinked a hypotonic lysate of Raji cells with DSSO before solubilizing the membrane fraction with 0.5% DOC lysis buffer and performing a pull-down enrichment using anti-CD79a, as described in the Methods section. Samples were separated by gel electrophoresis and visualised by Coomassie staining. In the bead eluate a band corresponding to the MW of CD79a was detected (Figure 8, Band F) along with five other higher-molecular-weight bands, consistent with crosslinked proteins (Figure 8, bands A–E). In-gel trypsin digestion was performed on these six bands and samples were prepared for mass spectrometry.

A False Discovery Rate (FDR) limit of <0.01 (High) and FDR of <0.05 (Medium) was utilized to identify the proteins present in each gel slice (Supplementary Data File). Previous MS analysis of WCLs had failed to detect any of the three components of the BCR (CD79a: Accession P11912; CD79b: Accession P40259; IGHM: Accession P01871; However, all three components were detected in the cross-linked sample enriched by pull down with anti-CD79a (Table 1 and Table S3). Band F, excised at the level of the 50 kDa MW marker, was expected to be enriched for uncross-linked CD79a. MS analysis detected 238 proteins in this band and, as expected, of the BCR components, only CD79a was present. All three components of the BCR were detected in bands B, D and E, while only IGHM was detected in the highest MW band, Band A (Table 1 and Table S3).

## 4. Discussion

### 4.1. Unmet Need for Proteomic Analysis of the BCR

Knowledge of the conformation of the BCR complex and its interactions with other proteins is critical to understanding its role in driving B cell malignancies and identifying potential therapeutic targets.

The BCR has been challenging to analyse, due to the difficulty of isolating and enriching this membrane-bound protein complex. There are approximately 120,000 BCRs on the B cell surface [21,22]; however, depending on the B cell activation state, there may be hundreds-of-millions to billions of proteins in a B cell [23]. Previously described proteomic strategies have included protein-G and protein-A purification steps, but have enriched for mIg and not the whole BCR complex [24]. Furthermore, isotypes other than IgG are not bound by protein-A and protein-G beads [25,26]. Consequently, the lack of MS workflows that use enriched, purified membrane-bound proteins to study structural and protein interactions has restricted the study of the BCR. Traditional methods to study the BCR have included X-ray crystallography, and cryogenic electron microscopy (cryo-EM) [3,27,28,29]; however, these provide limited structural, static data. There is a need to apply advanced proteomics to BCR analysis, including application of functional techniques to study the dynamic interactions of this receptor in vivo. The application of these techniques has been hampered by the inability to enrich the BCR effectively. The value of this reported technique is the extent to which the BCR can be enriched for future proteomic applications to discover receptor partners and functions.

There are several advanced proteomics techniques that can be employed with BCR enrichment methods to further study BCR-protein interactions and functions. For example, proximity labelling techniques such as BioID (biotin identification) or ascorbate peroxidase-catalysed proximity labelling can be coupled with BCR enrichment to identify proximal and interacting partner proteins with the BCR signalling complex or microenvironment [30,31]. Another example of the utility of our technique is evident with chemical cross-linking mass spectrometry (XL-MS). Cross-linking combined with BCR enrichment allows for stabilization of transient protein–protein interactions, enabling the identification of interacting partners that may dissociate during sample preparation or analysis [32,33]. The BCR enrichment product can be interrogated against protein microarrays containing a diverse array of proteins, enabling high-throughput screening for potential interaction partners or epitope mapping of BCR specificity [34]. Finally, functional proteomic assays, such as kinase activity profiling or phosphoproteomic analysis, can be performed in combination with BCR enrichment to assess the functional consequences of BCR signalling activation or modulation, facilitating mechanistic studies of B cell activation and immune regulation [35]. Ultimately, these advanced proteomic techniques assist in the interrogation of novel protein-interaction pathways, particularly in malignant B cell states with the potential to determine critical targets that confer pathogenicity to neoplastic clones, thereby identifying therapeutic targets.

This paper describes a method for enriching the BCR complex in sufficient quantities for harvest and further proteomic analyses described above. A potential weakness of the method is the number of cells required from human subjects to enrich proteins for proteomic methods. For the BCR enrichment studies, 25 × 10^6^ cells were required that would require collecting 25 mL of blood from a normal donor with a B cell count of 1 × 10^6^/mL. However, in B cell leukaemias such as chronic lymphocytic leukaemia the malignant B cell concentration in the blood may be as high as 200 × 10^6^/mL.

### 4.2. How Our Study Is Relevant and Meets Gaps in the Literature

The main objective for developing this pull-down process for the BCR complex was to establish an MS-compatible enrichment technique that did not interfere with BCR structure and function, unlike enrichment methods using biotinylation or radioactive tags, or peptide tags. A considerable number of the techniques mentioned above are integrated with MS, and hence our BCR enrichment technique was aimed at creating a product that was MS-compatible. Finally, to demonstrate the potential of our BCR enrichment method, we opted for XL-MS for preliminary investigations on the enriched product.

Targeting CD79a with a murine anti-human CD79a IgG1 antibody was the most effective strategy to pull down the whole BCR complex. Protein G beads have the highest affinity for mouse IgG1 antibodies [26,36,37], which explains the effectiveness of this pull-down method. Although there was pull down of both CD79b and IgM using the anti-CD79a antibody, the presence of unbound components of the BCR complex in the wash supernatant was consistent with separation of the components of the non-cross-linked BCR.

The optimal pulldown antibody: lysate ratio was 5 µL of anti-CD79a to 500 µg membrane fraction and the enrichment technique was further improved by stringent washing and incubating the antibody with the beads first, before mixing with the whole-cell lysate. The effectiveness of this sequence compared to incubating the whole-cell lysate with antibodies first followed by addition of beads may be that non-specific binding of proteins to the protein G beads in the latter sequence may reduce the sites available for occupation by the pull-down antibody. Finally, the optimal concentration for the MS-compatible detergent DOC was determined as 0.5%.

The advantages of this method include pulling down unmodified BCRs of all immunoglobulin isotypes. However, it is likely that further enrichment will be required prior to MS analysis of primary B cells because of the low abundance of the membrane-bound complex [24]. Moreover, to effectively integrate a method like XL-MS with BCR enrichment for uncovering protein-binding partners associated with the BCR, a broader range of variables requires optimization. This entails refining the choice of cross-linker, performing off-line enrichment of the cross-linked fraction, and optimizing both the parameters of the MS instrument and the data analysis techniques. These optimization variables are outside the scope of this current study, which focusses on the BCR enrichment process itself.

## 5. Conclusions

Whereas soluble Ig isotypes have been characterized using MS methods, mIg isotypes, due to relatively low abundance, have been difficult to study using advanced proteomic techniques [32]. Current enrichment strategies often modify the BCR from its endogenous state with the use of either transfected cell lines, recombinant proteins or tags before transient protein interactions can be captured.

An XL-MS combined with BCR enrichment using a specific MS1-3 workflow is currently being developed. For the preliminary data that are included in the paper, we used a 1D LCMS method which is identification focused, not quantitative. The control for our system was BSA which functioned as our system suitability test that demonstrated the LCMS was running at the specification. For future experiments combining BCR enrichment with XL-MS, a negative control such as a decoy antibody with our cell lysate will be used and checked for enrichment of the BCR product in the sample compared to the control.

There is an unmet need to improve techniques to enrich and isolate the BCR for advanced MS techniques. Ideally, a method is required that has the potential to minimally alter the weak, often transient interactions with the BCR and its partner proteins. This study describes a method to directly pull down the BCR of potentially all Ig classes that can be used to enrich the BCR for advanced proteomics, including XL-MS.

## Figures and Tables

**Table 1:** Summary of the characteristics of each gel band.

	Approx MW of Excised Gel Band (kDa)	No. of Proteins Detected in the Excised Gel Band	BCR Component Present		
			CD79a (P1192)	CD79b (P40259)	IGHM (P01871)
Band A	>160	176	No	No	Yes
Band B	130	561	Yes	Yes	Yes
Band C	80	428	No	Yes	Yes
Band D	70	525	Yes	Yes	Yes
Band E	60	609	Yes	Yes	Yes
Band F	50	238	Yes	No	No

## Data Availability

Data are contained within the article and Supplementary Materials.

## Supplementary Materials

The following supporting information can be downloaded at: https://www.mdpi.com/article/10.3390/cancers16132341/s1, Figure S1: Expression of BCR components by the aji cell line; Figure S2: Optimization of primary antibody dilution for Western blot; Figure S3: Application if the BCR enrichment protocol in the OSU cell line; Figure S4: Optimising anti-CD79a concentration for pull down; Figure S5: Optimisation of ash method for anti-CD79a antibody pull down in membrane fractions; Figure S6: Sequence of pull down enrichment process; Figure S7: Determining the optimal concentration to solubilise the membrane fraction for anti-CD79a pull down; Table S1: MS Instrument settings; Table S2: Detailed parameters for processing workflow. Table S3: Full list of peptides identified.
