# Supplementary material for: Method for B Cell Receptor Enrichment in Malignant B Cells
Source: Cancers (Basel). 2024 Jun 26;16(13):2341. doi: 10.3390/cancers16132341 (PMC11240526; doi:10.3390/cancers16132341)
Supplement: Supplementary file 1 [file cancers-16-02341-s001.zip › Supplementary Material.pdf]

Supplementary Figures and Tables

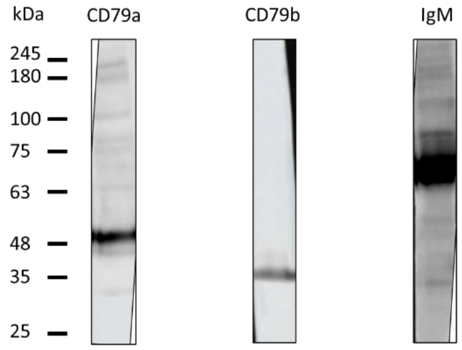

Figure S1. Expression of BCR components by the Raji cell line. The CD79a band was detected at approximately 48 kDa. The CD79b protein was detected at approximately 35 kDa. The IgM protein was detected between the 63 and the 75 kDa markers.

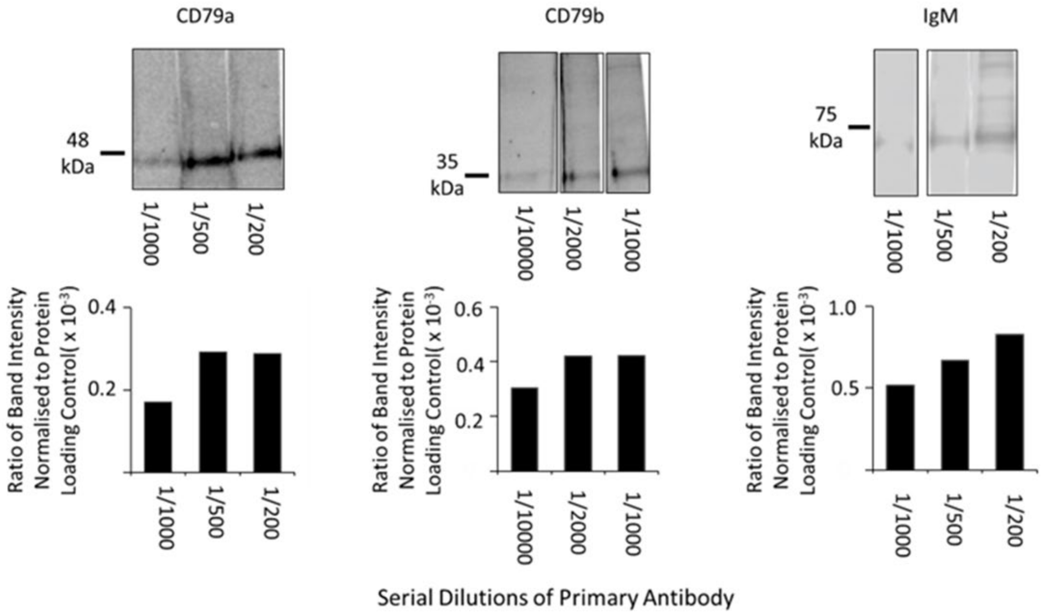

Figure S2. Optimization of primary antibody dilution for western blot. Anti-CD79a and anti-CD79b showed the highest sensitivity at 1/500 dilution and 1/1000 dilution respectively. For anti-IgM, a 1/500 dilution was optimal for band intensity while minimising background staining of non-specific bands

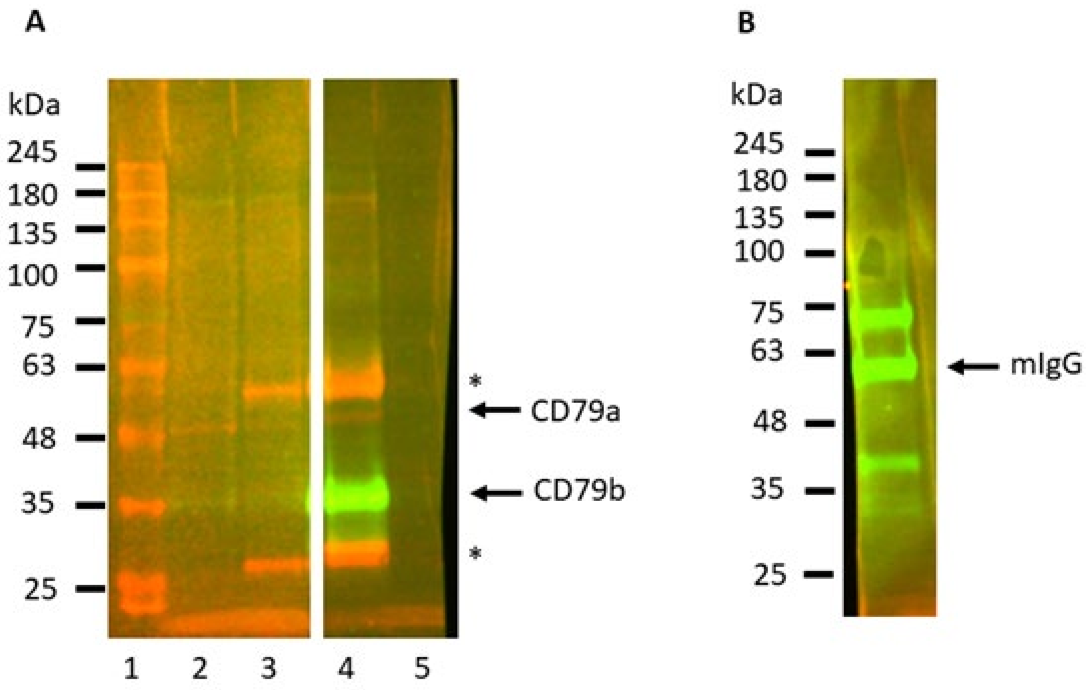

Figure S3: Application of the BCR enrichment protocol in the OSU Cell Line. **A.** membrane fraction of OSU was prepared and anti-CD79a was used in a pull-down assay as described in the methods. Input samples, bead eluate and bead supernatants were separated by SDS-PAGE and a blot was prepared. A) One part of the blot was probed for CD79a and CD79b using NIR techniques described in the methods. Lane 1: MW marker; Lane 2: Membrane Fraction; Lane 2: Membrane fraction plus anti-CD79a; Lane 4: Bead eluate; Lane 5: Bead Supernatant. Arrows indicate the band corresponding to CD79a (red band) and CD79b (green band). * Denotes the heavy and light chains of the pulldown antibody. CD79a and CD79b were enriched in the bead eluate (Lane 4) and were depleted from the bead supernatant (Lane 5). **B.** OSU membrane fraction probed with rabbit anti-human IgG. A band at molecular weight 52kDA corresponding to IgG is shown by the arrow. Non-specific bands are visible.

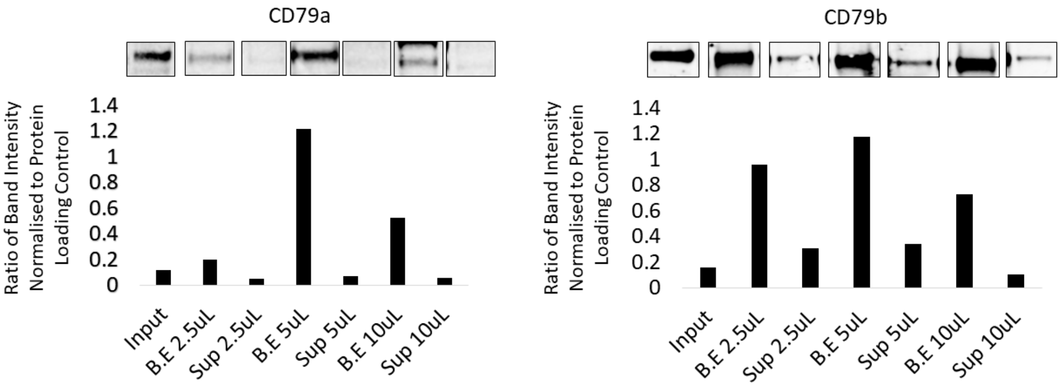

Sample	Band intensity*
Input	0.12
Bead Eluate 2.5 μL	0.2
Bead Supernatant 2.5 μL	0.05
Bead Eluate 5 μL	1.22
Bead Supernatant 5 μL	0.08
Bead Eluate 10 μL	0.53
Bead Supernatant 10 μL	0.06

Sample	Band intensity*
Input	0.16
Bead Eluate 2.5 μL	0.96
Bead Supernatant 2.5 μL	0.31
Bead Eluate 5 μL	1.18
Bead Supernatant 5 μL	0.35
Bead Eluate 10 μL	0.73
Bead Supernatant 10μL	0.11

Figure S4. Optimising anti-CD79a concentration for pull down. Sequential volumes of anti-CD79a pull down antibody were incubated with a standard mass (500 μg) of membrane fraction. CD79a, and CD79b were identified using western blot in the membrane fraction input, bead eluate, and supernatant following pull down. Bar charts (middle row) show the ratios of western blot band intensities normalized to protein loading control for CD79a and CD79b. Numerical values of the ratios represented in the bar charts are shown in tabular form below each chart. Abbreviations: Input, membrane fraction input; B.E, bead eluate; Sup, bead supernatant. *Ratio of band intensity normalised to protein loading control.

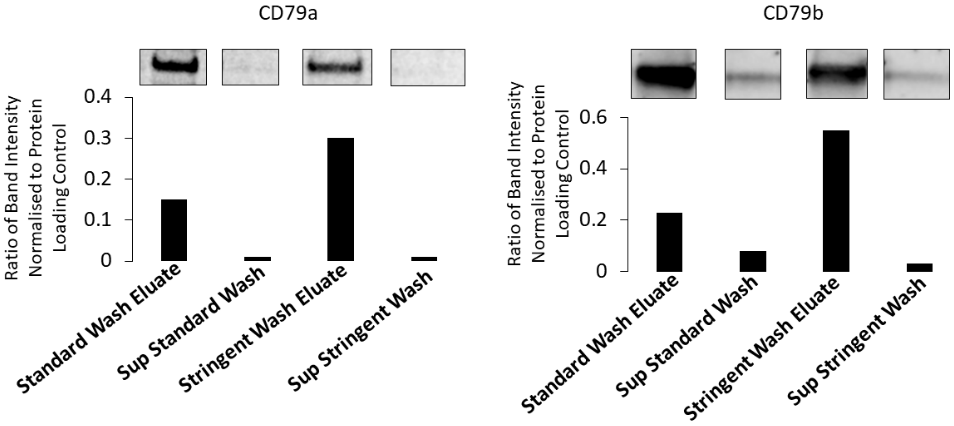

Figure S5. Optimisation of wash method for anti-CD79a antibody pull down in membrane fractions. CD79a (left panel) and CD79b (right panel) were identified using western blotting. Bar charts (lower row) show the ratios of western blot band intensities normalized to protein loading control for CD79a and CD79b. Western blot images correspond to labelling on bar charts. Abbreviations: Sup, supernatant.

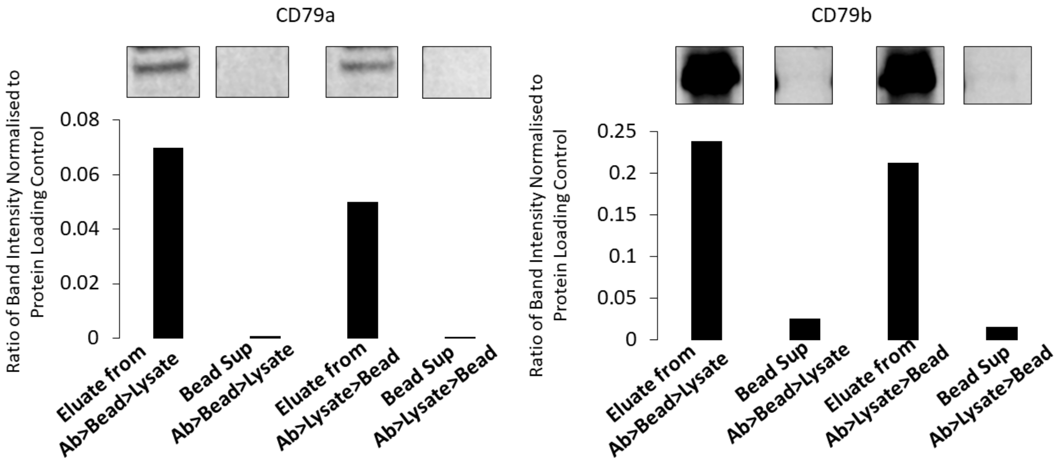

Figure S6 Sequence of pull-down enrichment process. Incubating the WCL with antibodies followed by addition of beads compared to applying antibodies to beads followed by WCL. CD79a (left panel) and CD79b (right panels) were identified using western blotting. Bar charts (lower row) show the ratios of western blot band intensities normalized to protein loading control for CD79a, and CD79b. Western blot images correspond to labelling on bar charts. Abbreviations: Ab, antibody; Sup, supernatant.

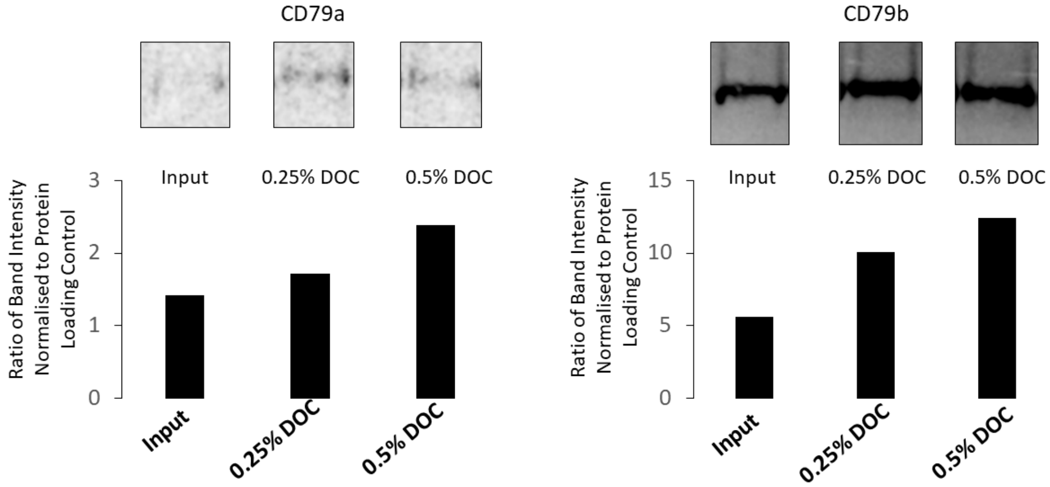

Figure S7. Determining the optimal DOC concentration to solubilise the membrane fraction for anti-CD79a pull down. The ratio of band intensity normalised to protein loading control for CD79a and CD79b identified using western blotting after pull down using anti-CD79a from membrane fractions solubilised in 0.25% 0.5% DOC buffer were compared. Abbreviations: DOC, sodium deoxycholate.

A) Methods Settings

**Global Settings**	
Use lock Masses	Off
Chrom peak width (FWHM)	15s
**Time**	
Method Duration	85 min

B) Experiment Settings

Runtime	0 to 85 min
Polarity	positive
In-source CID	0.0 eV
Default charge state	2
**Full MS**	
Microscans	1
Resolution	60000
AGC target	3e6
Maximum IT	20 ms
Number of scan ranges	1
Scan range	300 to 1650 m/z
Spectrum data type	Profile
**dd-MS2/dd-SIM**	
Microscans	1
Resolution	15000
AGC target	1e5
Maximum IT	28 ms
Loop count	15
MSX count	1
TopN	15
Isolation window	1.4 m/z
Isolation offset	0.0 m/z
Scan range	200 to 2000 m/z
Fixed first mass	100.0m/z
(N)CE/stepped (N)E	Nce:27
Spectrum data type	Profile
**dd Settings**	
Minimum AGC target	1.00e4
Intensity threshold	3.5e5
Apex Trigger	4 to 7 s
Charge exclusion	Unassigned, 1,5-8, >8
Multiple charge states	all
Peptide match	preferred
Exclude isotopes	on
Dynamic exclusion	30.0 s
If idle…	Do not pick others

C) LC Gradient

Time		
-0.100 [min]	Equiibration	Sampler.InjectValveToLoad
		PumpModule.NC_Pump.Flow.Nominal:0.450[ul/min]
		PumpModule.NC_Pump. %B.Value: 3[%]

		PumpModule.NC_Pump.Curve: 5
0.000 [min]	Inject Preparation	PumpModule.NC_Pump.Flow.Nominal:0.450[ul/min]
		Wait Sampler.Ready And PumpModule.LoadingPump.Ready And PumpModule.NC_Pump.Ready And ColumnOven.Ready
0.000 [min]	Inject	Sampler.Inject
0.000 [min]	Start Run	PumpModule.NC_Pump.NC_Pump_Pressure.AcqOn
0.000 [min]	Run	PumpModule.NC_Pump.Flow.Nominal:0.450[ul/min]
		PumpModule.NC_Pump. %B.Value: 3[%]
		PumpModule.NC_Pump.Curve: 5
0.100 [min]		PumpModule.NC_Pump. %B.Value: 5[%]
		PumpModule.NC_Pump.Curve: 5
14.000 [min]		PumpModule.NC_Pump. %B.Value: 5[%]
		PumpModule.NC_Pump.Flow.Nominal:0.450 [ul/min]
		PumpModule.NC_Pump.Curve: 5
14.100 [min]		PumpModule.NC_Pump.Flow.Nominal:0.30 [ul/min]
74.100 [min]		PumpModule.NC_Pump.Flow.Nominal:0.300 [ul/min]
		PumpModule.NC_Pump. %B.Value: 30 [%]
		PumpModule.NC_Pump.Curve: 5
78.000 [min]		PumpModule.NC_Pump.Flow.Nominal:0.300 [ul/min]
		PumpModule.NC_Pump. %B.Value: 60 [%]
		PumpModule.NC_Pump.Curve: 5
82.000 [min]		PumpModule.NC_Pump.Flow.Nominal:0.30 [ul/min]
		PumpModule.NC_Pump. %B.Value: 98 [%]
		PumpModule.NC_Pump.Curve: 5
85.000 [min]		PumpModule.NC_Pump.Flow.Nominal:0.300 [ul/min]
		PumpModule.NC_Pump.Curve: 5
		PumpModule.NC_Pump. %B.Value: 98 [%]
85.000 [min]	Stop Run	PumpModule.NC_Pump.NC_Pump_Pressure.AcqOff

Table S1: MS Instrument settings

Processing Node 0	1. Search Settings	Protein Database: human_uniprot_May2020.fasta<br>Enzyme Name: Trypsin (full)<br>Precursor Mass tolerance: 20ppm<br>Fragment Mass Tolerance: 0.5Da
	2. Regression Settings:	Regression model: Non-linear regression<br>Parameter tuning: Coarse
	3. General Settings	Precursor Selection: Use MS1 precursor<br>Use Isotype Pattern in Precursor Reevaluation: True<br>Provide Profile Spectra: Automatic
	4. Spectrum Properties Filter:	Lower RT limit: 0<br>Upper RT limit: 0<br>First Scan: 0

Processing Node 1:<br><br>Spectrum Selector		Last Scan: 0<br>Lowest Charge State: 0<br>Highest Charge State: 0<br>Min. Precursor Mass: 350Da<br>Max Percursor Mass: 5000Da<br>Total Intensity Threshold: 0<br>Minimum Peak Count: 1
	5. Scan Event Filters:	MS order: Is not MS1<br>Min. Collision Energy: 0<br>Max. Collision Energy: 1000<br>Scan type: Is Full
	6. Peak Filters	S/N Threshold (FT-only): 1.5
	7. Replacements for Unrecognised Properties:	Unrecognized Charge Replacements: Automatic<br>Unrecognized Mass Analyzer Replacements: ITMS<br>Unrecognized MS Order Replacements: MS2<br>Unrecognized Activation Type Replacements: CID<br>Unrecognized Polarity Replacements: +<br>Unrecognized MS Resolution@200 Replacements: 60000<br>Unrecognized MSn Resolution@200 Replacements: 30000
	8. Precursor Pattern Extraction	Precursor Clipping Range Before: 2.5Da<br>Precursor Clipping Range After: 5.5Da
Processing Node 2:<br><br>Mascot (Vr 2.8)	9. Input Data	Instrument: Default<br>Protein Database: human_uniprot_May2020.fasta<br>Enzyme Name: Trypsin<br>Maximum Missed Cleavage sites: 2<br>Taxonomy: All entries<br>Error Tolerant Search: False
	10. Tolerances	Fragment Mass Tolerance: 0.05Da<br>Precursor Mass Tolerance: 10ppm<br>Use Average Precursor Mass: False
	1. Dynamic Modifications:	Show All Dynamic Modifications: False<br>1. Dynamic Modification: Acetyl (Protein N-term)<br>2. Dynamic Modification: Deamidated (NQ)<br>3. Dynamic Modification: Carbamidomethyl(C)<br>4. Dynamic Modification: Oxidation (M)
Processing node 3:<br><br>Percolator	1. Target/Decoy Strategy:	Target/Decoy Strategy:<br>- Target/Decoy Selection: Concatenated<br>- Validation based on: q-value
	2. Input Data	Maximum Delta Cn: 0.05<br>Maximum Rank: 0
	3. FDR Targets	Target FDR (strict): 0.01<br>Target FDR (Relaxed): 0.05

Table S2: Detailed Parameters for Processing Workflow
